# Antifouling Mortars for Underwater Restoration

**DOI:** 10.3390/nano12091498

**Published:** 2022-04-28

**Authors:** Michela Ricca, Silvestro Antonio Ruffolo, Mauro Francesco La Russa, Concetta Rispoli, Celestino Grifa, Aranzazu Sierra-Fernández, Rafael Fort, Luciana Randazzo

**Affiliations:** 1 Dipartimento di Biologia, Ecologia e Scienze della Terra, Università della Calabria, Via Pietro Bucci, 87036 Arcavacata di Rende, Italy; michela.ricca@unical.it (M.R.); mlarussa@unical.it (M.F.L.R.); 2 Dipartimento di Scienze della Terra, dell’Ambiente e delle Risorse, University of Naples Federico II, Complesso Universitario Monte Sant’Angelo, ED. 10, Via Cintia 26, 80126 Naples, Italy; concetta.rispoli@unina.it; 3 Dipartimento di Scienze e Tecnologie, Università degli Studi del Sannio di Benevento, Via De Sanctis snc, 82100 Benevento, Italy; celgrifa@unisannio.it; 4 Instituto de Geociencias (CSIC, UCM) C/Severo Ochoa 7, 28040 Madrid, Spain; arsierra@ucm.es (A.S.-F.); rafael.fort@csic.es (R.F.); 5 Dipartimento di Scienze delle Terra e del Mare, Università di Palermo, Via Archirafi, 26, 90123 Palermo, Italy; luciana.randazzo@unipa.it

**Keywords:** magnesium hydroxide, mortars, submerged sites, biofouling, geomaterials, restoration, nanoparticles

## Abstract

This research has focused on the assessment of the compositional features and mechanical and antifouling performances of two different mortars formulated for an underwater setting, and which contain Mg(OH)_2_ as an antifouling agent. Regarding the mechanical characterization, the uniaxial compressive strength and flexural strength were measured. The composition of the materials was explored by differential thermal/thermogravimetric analysis (DTA-TG), X-ray diffraction analysis (XRPD), and scanning electronic microscopy (SEM) coupled with EDS microanalysis. The assessment of the biological colonization was evaluated with colorimetric analysis and image analysis. The results suggest that both mortars have good mechanical resistance once set underwater. Moreover, the adding of Mg(OH)_2_ improves the resistance toward biofouling; this was observed both in laboratory and sea-exposed specimens.

## 1. Introduction

In recent decades, there has been an increasing interest in the study of the degradation phenomena affecting the archaeological sites located in submarine environments [1,2] The most recent guidelines about underwater heritage are focused on in situ conservation (i.e., UNESCO Convention on the Protection of the Underwater Cultural Heritage, 2 November 2001). Since their issue, several techniques and materials have been proposed for their conservation [3,4,5,6,7]. The main cause of the decay of natural (e.g., stone) and artificial geomaterials (e.g., mortars, pottery) in an underwater environment is biodeterioration [8,9] in the form of biofouling and bioerosion phenomena [10,11,12,13]. Biological weathering, commonly called biofouling, is a consequence of physical, chemical, and biological factors that contribute to the decay of submerged materials. It is a natural process occurring on underwater items through the colonization and overgrowth of epilithic and endolithic organisms and represents a major economic problem in both archaeological sites and maritime industries [14]. The first events in biofouling formation are the deposition of multilayered organic matter (biofilm), followed by colonization with specific bacterial species (microfouling) [11]. This primary microbial film can prepare the surface for subsequent colonization by extracellular polymeric substances production, which are responsible for the adhesion and growth of new microorganisms, plants, algae, and sessile organisms. The materials used for underwater restoration may be prone to rapid biodeterioration as well, and among them, mortar-based materials are common in the conservation of underwater archaeological patrimony. In order to be suitable for underwater application, a good hydraulic behaviour and mineralogical stability are required for restoration mortars [15]; moreover, a low susceptibility to bio-colonization is expected during their formulation.

In this study, attention was paid to magnesium hydroxide Mg(OH)_2_ as an antifouling agent [16] in different mortar pastes. This material had shown a promising biocidal activity when used in coatings for stone materials, especially in the form of nanoparticles with dimensions ranging from ~30 and ~450 nm and well-defined hexagonal shapes [17,18,19,20]. Magnesium-based materials have generated considerable interest as antimicrobial agents in a wide variety of applications, such as in the treatment of stone materials used in cultural heritage [19,21,22]. In particular, the use of these nanostructured inorganic materials has proved to be a highly effective means to prevent bio-colonization as well as to carry out a consolidating action. Commonly, nanostructured materials possess specific physicochemical properties that, in combination with their large surface area and dimensions, allow them to interact and internalize within cells, displaying a broad spectrum of antibacterial activity [23,24]. Moreover, their modular nature means that a library of relatively low-cost materials with different sizes, shapes, surface properties, and chemical compositions can be developed, leading to a great potential for developing effective antimicrobial agents with high stability under harsh environmental conditions. It is important to note that the antibacterial effect often depends on the size, shape, chemical composition, and surface properties (e.g., hydrophobicity) of the nanoparticles [22,25,26].

The behavior of these formulations in terms of mechanical strength, compositional characteristics and resistance to biodeterioration when exposed to the underwater environment was evaluated in two different exposure conditions (i.e., in lab and in situ trials).

## 2. Materials and Methods

For the experiment, two types of natural hydraulic mortars were selected: (i) one is based on Volteco Microlime Gel (Volteco, Ponzano Veneto, Italy) (hereinafter named VM), a mixture composed of aerial and hydraulic binders loaded with organic thickeners which provide pseudoplastic behavior; (ii) the second is based on NHL 3.5 Saint Astier (CESA, Saint-Astier, France) (hereinafter named SA), 3.5 type natural hydraulic lime (NHL).

**Figure 1:**
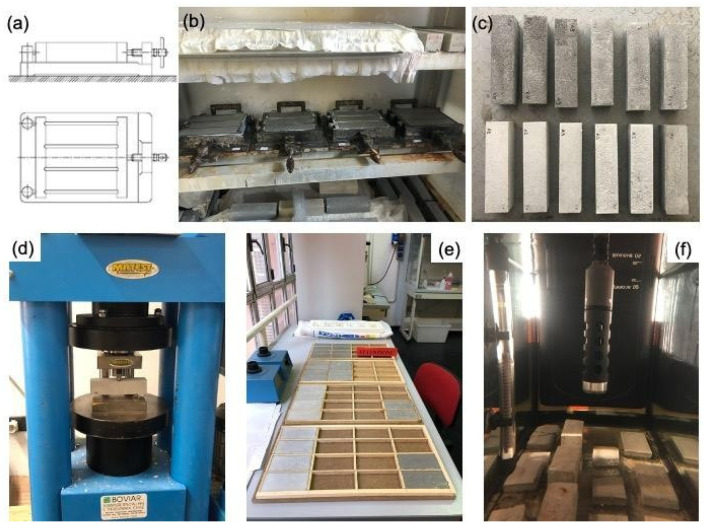
(**a**) Steel mold scheme consisting of three horizontal compartments for prismatic specimens’ preparation (16 × 4 × 4 cm); (**b**) cabin storage for curing specimens; (**c**) specimens after 28 days of curing time; (**d**) compressive strength testing machine; (**e**) wood mold for specimen preparation (10 × 8 × 0.8 cm); (**f**) rectangular glass tank for lab experimentation.

**Figure 2:**
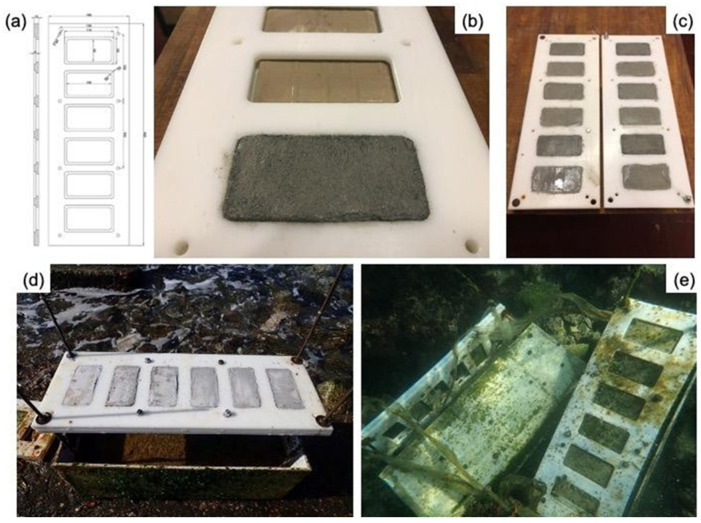
(**a**–**c**) Ertacetal holder plates with six (6) rectangular compartments for housing the mortar specimens (10 × 5 × 0.8 cm) and maintaining them fixed into the open sea bottom; (**d**) concrete anchors with the Ertacetal holders positioned before immersion; (**e**) view of the holders into the sea bottom.

A pozzolanic aggregate was mixed in both starting materials; for this purpose, the Pozzolana fine (Rime 1, Srl, Rome, Italy) was added, with a particle size from 75 microns to 1 mm. The mortars were prepared with a binder/aggregate ratio equal to 1/2 and a water amount of 30% *w*/*w* with respect to the binder amount. To explore the use of magnesium hydroxide (Mg(OH)_2_) nanoparticles as antifouling agents for mortars, 44 specimens were mixed with 1% *w*/*w* magnesium hydroxide Mg(OH)_2_ nanoparticles, synthesized according to Sierra-Fernandez et al., 2019. A total of 22.02 g of magnesium methoxide (Mg(OCH_3_)_2_ was dissolved in ethanol (85.2 mL) at 60 °C. After that, 25.2 mL of deionized water was added to the above solution drop-wise and allowed to reflux at 60 °C for 24 h. Thereafter, the solution was centrifuged and washed several times with mixtures of ethanol: distilled water. Finally, the sample was dried under an inert gas atmosphere at 100 °C for 4 h. Two series of specimens were prepared to evaluate the performance of artificial stone materials before and after exposure to seawater, under different exposure conditions. Respectively, the samples were of dimensions 16 × 4 × 4 cm for mechanical tests and 10 × 5 × 0.8 cm for the evaluation of biological growth. Before the exposure of the mortars to the different experimental conditions, a curing time of 28 days was achieved. Specifically, blends were poured into molds and then were transferred to a cabin storage after 24 h, where they were kept under controlled conditions of 20 ± 2 °C, and relative humidity >95% for 28 days. A summary of the specimens used in the two experimental sets, with a brief description and analytical techniques used, are shown in Figure 1 and Figure 2 and summarized in Table 1.

For the simulation of underwater condition, the specimens were settled down in a rectangular glass tank (aquarium) of 100 L capacity (100 cm long, 45 cm deep, 45 cm wide) filled with 80 L of natural seawater taken at a depth of about 2 m and filtered to remove particles. A sandy substrate inside the aquarium was collected from a clean site nearby the same area of the in situ experiment, near the archaeological site of Castrum Novum (Santa Marinella, Rome), a Roman colony whose ruins are located between Torre Chiaruccia and Casale Alibrandi (Lazio, Italy) [27,28]. The aquarium was left to stabilize for about a month with constant monitoring of chemical–physical parameters via a YSI 556 MPS multiparametric probe (YSI, Yellow Springs, OH, USA). After the stabilization period, mortars samples, with and without nanoparticle additions (control groups), were immersed in the aquarium for 12 months. During this time, physical and chemical parameters (i.e., temperature, pH, oxygen level, salinity and resistivity) were monitored monthly (Table 2).

At the same time, in order to assess the biological colonization, specimens underwent a field exposure test at the fishpond located in the archaeological site of Castrum Novum. The underwater site was chosen as a case study for the present research, where several mortar fragments belonging to different archaeological fishpond structures were recovered and characterized from a textural, mineralogical, and geochemical point of view [12]. The acquired dataset has been useful for the new restoration mortars designed for the present research.

As regards the mechanical characterization, the uniaxial compressive strength and the flexural strength of the mortars were determined according to the relevant European standards [29,30] by using a compression and flexural machine 3000 kN semi-automatic, Digitec, Matest, model C070, on prismatic specimens with dimensions of 40 × 40 × 160 mm. The latter were performed only on the samples exposed in the tank. All the values were obtained as the average of three tested specimens.

In order to characterize the composition of the mortars and its evolution within the setting, differential thermal analysis (DTA) in combination with thermogravimetric (TG) analysis was carried out by using a STA TG-DSC instrument (NETZSCH STA 449 F3 Jupiter. The samples (100 mg) were heated from 20 °C to 1050 °C, with a heating rate of 10 °C/min in a nitrogen atmosphere (flow rate 60 mL/min). TG and DSC curves were acquired and subsequently processed with the NETZSCH Proteus 6.1 Software.

Mineralogical phases were detected by diffractometric analyses, which were carried out using a Bruker D2 A Bruker D2 Phaser (LINKEYE detector) diffractometer with the following operating conditions: CuKα radiation, 30 kV, 10 mA, 2ϑ range from 4 to 70°, 0.02 2ϑ step size, 120 s per step counting time.

Each sample was disaggregated by hand in an agate mortar to obtain a homogeneous powder (particle size < 200 μm). An amount of 20 wt.% corundum (α-Al_2_O_3_, Buehler micropolish, 1 μm grain size) was added as an internal standard. This mixture was subsequently micronized (grain size < 10 μm) using a McCrone Micronising Mill with agate cylinders and 10 mL of deionized water for 15 min of grinding time. For the qualitative interpretations of XRPD patterns, the Panalytical HighScore Plus 3.0 d software was used, whereas the BRUKER TOPAS 5.0 software was employed for quantitative evaluations with the combined RIR/Rietveld approach (Chung, 1974; Bish and Post, 1993).

Morphological observations on a microscale, aiming to determine material characterization as well as the biological degradation that developed on the surface of the immersed experimental specimens, were carried out by scanning electron microscopy (SEM). For this purpose, a Zeiss EVO 15 HD VPSEM microscope was used with an electron voltage of 20 kV; in addition, microchemical analyses were performed by Oxford XmaX 80 Energy- dispersive X-ray spectroscopy (EDS).

In order to quantify the biological growth developed on the specimens immersed in laboratory tank, colorimetric and image analyses of the surfaces were carried out. For colorimetric measurements, a CM- 2600 d Konica Minolta spectrophotometer was used. Chromatic values are expressed according to the CIE (Commission Internationale d’Eclairage) L*a*b* space, where L* is the lightness/darkness coordinate, a* the red/green coordinate (+a* indicating red and −a* green), and b* the yellow/blue coordinate (+b* indicating yellow and −b* blue) [31]. Measurements were carried out using an 8.0 mm-diameter viewing aperture, specular component excluded (SCE), UV 0%, Illuminant D65 and 10° observer angle. The color changes were expressed in terms of ΔE:

∆E = √∆L*^2^ + ∆a*^2^ + ∆b*^2^ (1)

All the given results are average values of 10 measurements taken on each specimen (10 on each specimen) at different stages of exposure.

Regarding image analysis, high-resolution pictures were acquired by means of a scanner and processed with the ImageJ software, which is based on Sun-Java and developed by the US National Institutes of Health [32] (Collins, 2007). Collected images first underwent a preprocessing protocol, then, an average image was generated from the set of 18 sub-images of blank specimens (before exposure). After that, from each picture of exposed mortar (3 sub-image for each type), the percentage of coverage of biological colonization was calculated.

## 3. Results and Discussions

The data will be discussed separately, considering the experimentation performed both in the lab and at the underwater pilot site.

Table 3 reports the output data of flexural strength and uniaxial compressive strength for mortars with and without Mg(OH)_2_ nanoparticle additions. The uniaxial compressive strength was measured after 28 days of curing time and 200 days after seawater immersion. Results suggest that the adding of Mg(OH)_2_ had a slight effect on the mechanical resistance of the mortars, since for all tests, there was a slight decrease in the values. This effect is probably due to the fact that Mg(OH)_2_ does not have any active role in the formation of chemical bonds, it has no reactivity, and it is just an inert component. Moreover, as will be shown later by TGA, the mortar samples with Mg(OH)_2_ nanoparticle additions and exposed to in-situ conditions presented a higher degree of hydration, which inhibited the carbonation rate. Therefore, the lower rate of carbonation detected in these samples together with the presence of hydrated phases with deficient stability could be an important factor explaining this slight reduction in their strength development. Additionally, there is no clear variation of the compressive strength over time; there is an increase in all values after 200 days, although they have the same magnitude of the standard deviations. With regards to the comparison between the prepared mortars and the values given by the producers, there is a slight deviation, in particular, the data is better for the SA mortar, whereas the VM values in terms of compressive strength are comparable. This could be linked to an increased pozzolanic activity due to the further addition of pozzolans to the mortars, particularly in the SA typology.

Colorimetric analysis was carried out on samples after 28 days of immersion in laboratory tank, and after 200 days as well (Table 4). For all samples, just low chromatic variations were detected, however, the samples containing Mg(OH)_2_ showed a lower chromatic variation. The color change over time is mainly due to biological colonization; this suggests that Mg(OH)_2_ exerts an antifouling effect.

### In-Situ Experimentation

Table 5 it reports the mineralogical composition according to XRD analysis, showing all the specimens investigated both prior and after the exposure to sea water. There is a compositional difference between SA and VM mortars, maybe due to a different initial composition of the binder and the subsequent reactions between the binder and aggregate that occurred. Specifically, VM is characterized by a greater amount of mineralogical phases belonging to sandy aggregates such as quartz, plagioclase, pyroxene and leucite with respect to SA mortar. In addition, VM showed a relatively lower amount in calcite. Such values could affect the entity of the pozzolanic reaction that occurred in the mortars, which is related to the amount of amorphous phases (hydrated compounds such as C-S-A-H or hydrated calcium silicates and aluminates). From a performance point of view, the major development of neoformation mineralogical phases in SA, linked to pozzolanic reactions, would make the mortar more suitable for consolidation purposes, due to an increase in resistance and cohesion properties.

However, this seems to be disproved by mechanical measurements, since VM samples are slightly more resistant than SA ones. Such results could depend on the exposure site, i.e., from the tank and in situ field tests. Moreover, differences in the degree of carbonation reaction associated with the incorporation of nanoparticles in the samples could be established. In this way, a higher content of calcite was detected in samples without nanoparticles (Table 5). In contrast, mortars with Mg(OH)_2_ nanoparticle additions show the major content in amorphous phases after 12 months of exposition, since more hydrated phases could be formed in them. These results are in line with the following TGA findings, in which a higher amount of water content and hydrated phases (weight loss in region 40–200 °C) and a reduction in the temperature range attributed to the decomposition of carbonates (600–850 °C) were determined in these specimens. On the other hand, no noteworthy difference is evident with regards to the time of exposure to seawater.

Thermal analysis was performed on samples (with and without magnesium hydroxide at different stages of exposure, i.e., 3, 6 and 12 months) in a static air atmosphere, in the temperature range of 20–1000 °C, with a gradient of 10 °C/min. Usually, in the mortar characterization, the thermal transformations that occur during the heating of the sample are generally associated: (a) with a loss of hygroscopic water (T < 120 °C); (b) with a loss of crystallization water of hydrated salts, such as gypsum (120 < T < 200 °C) [33]; (c) with a loss of structurally bound water belonging to hydraulic components, such as hydrated calcium silicate (CSH) or hydrated calcium aluminates (CAH) (120 < T < 600 °C) [34,35]; and (d) with the decomposition of carbonates (600 < T < 900 °C) [33]. However, due to the different thermal stability of nanocrystals [36], and the fact that metastable calcium carbonate polymorphs and hydrated amorphous calcium carbonate are decomposed in the same temperature range (400–600 °C) [37], the interpretation of thermal analysis in the mortars can be more complex.

It was not possible to carry out the analytical sequence on all the types of formulated mortars. In particular, the data regarding VM (12 months), VM/Mg-3 months and 12 months are missing. This was due to strong storms that affected the archaeological site during the experiment period. As a result, some of the experimental mortars housed in the sample holder were swept away and it was not possible to recover them in any way.

**Figure 3:**
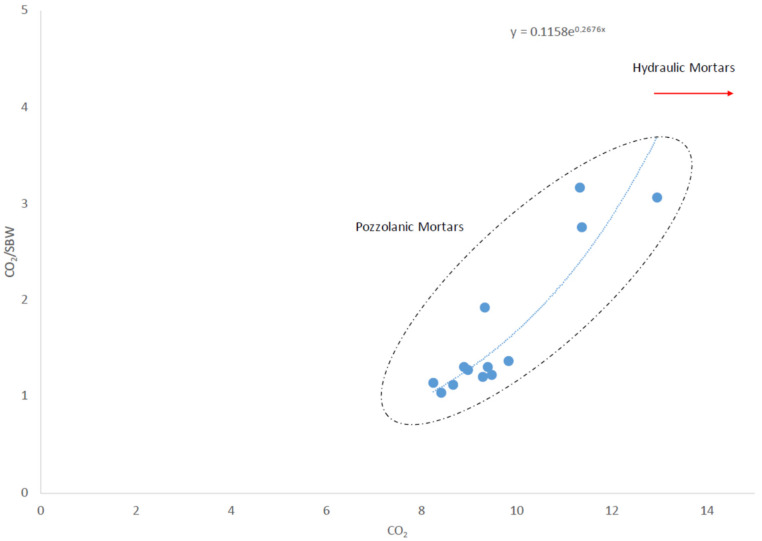
Hydraulic classification of mortars through the CO_2_/SBW ratio. CO_2_ refers to the loss in weight (%) in the range of 600–850 °C, and SBW (Structural Bound Water) refers to that in the range of 200–600 °C.

The recognition and quantitative evaluation of the thermally reactive phases of the aggregate and of the binder were achieved by thermogravimetric analyses (Table 6) that are considered a widely accepted approach for the hydraulic classification of ancient mortars [38,39]. In the first considered interval of temperature (40–200 °C), a weight loss (on average 3 wt%) due to dehydratation of non-chemically bonded water occurred, the following temperature range (200–600 °C) shows the thermal effects of the weight loss due to Structural Bound Water (SBW), generally related to the presence of hydrated compounds such as calcium silicate hydrate (CSH) and/or calcium aluminate hydrate (CAH); the weight loss ranges from 4.12 to 7.74 wt%. In the 600–850 °C the decomposition of calcite and other carbonates is the most prominent thermal effect (on average 10 wt%). The calculated stoichiometric calcite ranges from 18.73 to 29.37 wt%. Finally, at highest temperatures (>850 °C), the weight loss was ca. 1 wt%, likely attributable to halides component. DSC highlights that all thermal reactions are endothermic and considering the CO_2_/SBW vs. CO_2_ ratio (Figure 3), all the samples can be considered pozzolanic mortars [40]. Remarkably, the addition of Mg(OH)_2_ nanoparticles provoked, both in the mortars composed by a mixture of aerial and hydraulic binders loaded with organic thickeners (VM) and the specimens based on natural hydraulic lime (SA), a decrease in carbonation rate (Table 6). Different levels of carbonation between combinations could be due to the different levels of pozzolanic reactions. Thus, the lowest value corresponding to the carbonation rate (600–850 °C) belonged to mortar samples with nanoparticle additions and which were exposed to longer times (VM/Mg 6 months and SA/Mg 12 months). Moreover, these samples presented more weight losses in the range attributed to the water content (40–200 °C). In this way, after six (6) months of exposition, the sample VM with Mg(OH)_2_ nanoparticle addition (VM/Mg 6 months) experienced an increase of 17.1% in comparison with its analogue without nanoparticles (VM 6 months). Likewise, the mortars based on natural hydraulic binder (SA) with nanoparticle additions (SA/Mg 12 months) showed an increase of 10.9% in comparison with the specimens without nanoparticles (SA/12 months) (Table 6). This water content may come from hydrated silica originating from the CSH carbonation, making pozzolanic reactions dominate over the carbonation reaction in these specimens [41].

Scanning electron microscope observations were carried out on the surface portion of the samples analyzed to evaluate the effectiveness of Mg(OH)_2_ nanoparticles as additives with antifouling properties.

**Figure 4:**
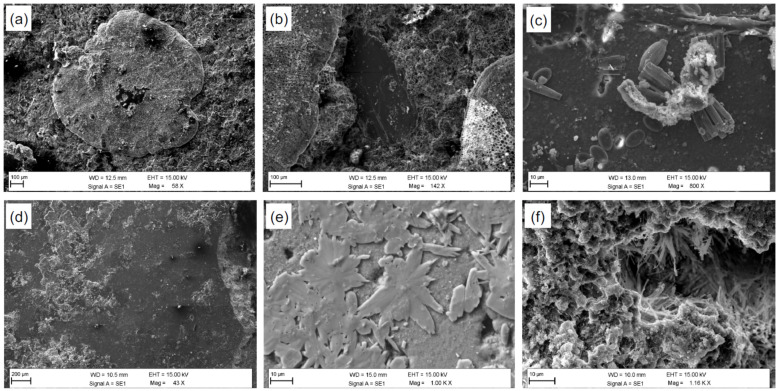
Representative SEM-BSE images of mortar specimens without (**a**–**c**) and with magnesium-based additives (**d**–**f**). Specifically: (**a**,**b**) deposits of crustose calcareous algae with honeycomb-like thalli; (**c**) diatoms; (**d**–**f**) new mineral phases, different in shape and size, covering the entire investigated area.

As can be seen from the images acquired and reported below (Figure 4), colonization shows similar characteristics regardless of the base mortar used for the formulation of the specimens. The only noteworthy differences are a higher presence of biological species found on the surface of the specimens without additives, compared to those with additives with Mg(OH)_2_ nanoparticles. The macrofouling growth on untreated mortars samples is primarily characterized by coralline algae showing calcareous honeycomb-like thalli along with diatoms contamination. On the contrary, the mortars with additives show the almost absence of biomass, with surfaces almost entirely covered by newly formed mineralogical phases.

**Figure 5:**
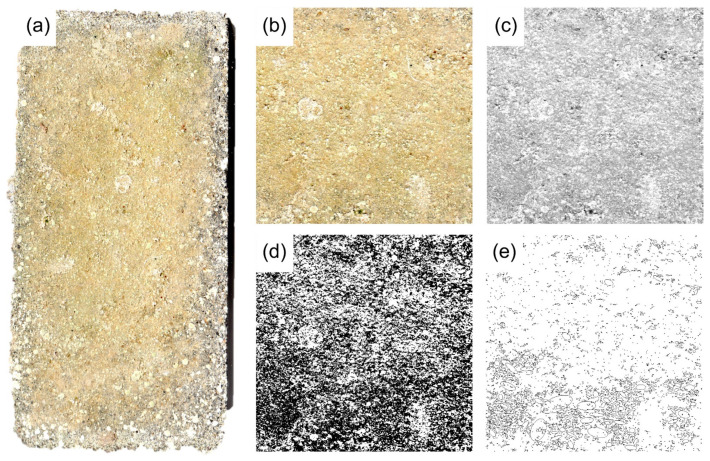
Images involved in the image analysis process for assessing the percentage of coverage. (**a**) Picture of the samples to be analyzed; (**b**) selected area; (**c**) reduction to an 8-bit image; (**d**) thresholding and subtracting process; (**e**) binary image ready to be analyzed.

**Figure 6:**
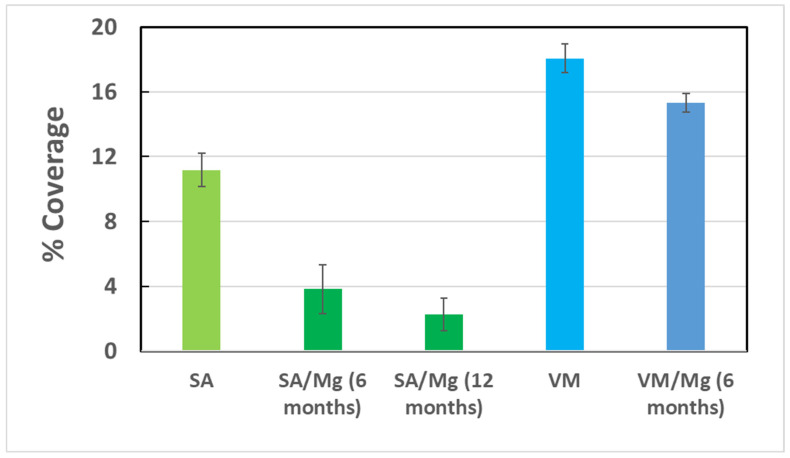
Summary of Coverage evaluation performed on sea-exposed samples.

An assessment of biological colonization of mortars exposed to real conditions was carried out by image analysis and summarized in Figure 5. Once collected and dried from seawater, samples were brushed to remove the incoherent material. The images of exposed surfaces were then scanned, and then an area was selected and transformed into an 8-bit image. After a thresholding and a subtracting process, the percentage of coverage was established for each image. The results of such calculations are summarized in Figure 6. There is a different behavior between SA and VM samples, since SA suffer less colonization than VM mortars; this could be due to the fact that VM contains a certain amount of organic matter which can play an active role in the colonization process. Regarding the adding of Mg(OH)_2_, results suggest that this material is effective in reducing biological colonization; this is in accordance with measurements carried out on laboratory-exposed samples and investigated by colorimetric and microscopic observations by SEM. SA/Mg samples exhibited better behavior against colonization.

## 4. Conclusions

This study assessed the compositional features and the mechanical performances of two different mortars formulated for underwater setting. The first one (named VM) was based on mixture composed of aerial and hydraulic binders, loaded with organic thickeners which provide pseudoplastic behavior, whereas the second (named SA) was based on natural hydraulic lime and pozzolanic aggregate. In addition, it tested the antifouling features for the mortars added with Mg(OH)_2_. Measurements were carried out on specimens exposed in a laboratory tank and in the sea, in real conditions. The results suggested that both mortars have good mechanical resistance once set underwater, although VM mortars seem to be more resistant than SA. The adding of Mg(OH)_2_ improves the resistance toward biofouling; this was observed both in laboratory and sea exposed specimens, in particular, SA formulation containing Mg(OH)_2_ showed the best performance.

## Figures and Tables

**Table 1:** Summary of the specimens used in the two experimental sets, with a brief description and analytical techniques used.

Sample Code	Lab Experiment		In Situ Experiment			
	Mechanical Test	Colorimetric Analysis	XRD	SEM/EDS	TG/DTA	Image Analysis
Size of Specimens (cm)	16 × 16 × 4	10 × 5 × 0.8	10 × 5 × 0.8 (*n* = 16)			
SA	x (*n* = 6)	x (*n* = 1)	x	x	x	x
VM	x (*n* = 6)	x (*n* = 1)	x	x	x	x
SA/Mg	x (*n* = 6)	x (*n* = 1)	x	x	x	x
VM/Mg	x (*n* = 6)	x (*n* = 1)	x	x	x	x

**Table 2:** Values of the chemical–physical parameters of water in the laboratory tank.

DateTime D/M/Y	Temperature °C	pH	TDS g/L	SpCond mS/cm	Salinity ppt	Resistivity KOhm.cm	ORP mV	DO% %	DO Conc mg/L	Cond mS/cm
21/11/18	22.82	8.20	35.05	53.93	35.68	0.02	304.78	70.07	4.91	51.68
12/12/18	20.50	8.13	35.46	54.55	36.17	0.02	254.56	28.47	2.07	49.86
10/01/19	19.70	8.29	35.65	54.85	36.40	0.02	202.68	190.06	14.02	49.29
07/02/19	22.94	8.12	36.17	55.65	36.96	0.02	204.50	20.27	1.41	53.46
06/03/19	23.60	8.14	36.16	55.63	36.94	0.02	206.40	92.47	6.35	54.15
03/04/19	23.11	8.14	35.41	54.48	36.09	0.02	240.86	2.04	0.14	52.51
04/05/19	23.12	8.12	35.46	54.56	36.15	0.02	177.29	2.04	0.14	52.60
10/06/19	22.45	8.05	35.43	54.51	36.12	0.02	221.79	33.31	2.34	51.85
11/07/19	27.56	7.91	35.90	55.23	36.54	0.02	155.48	16.05	1.03	57.93
01/08/19	27.67	7.91	36.02	55.41	36.68	0.02	154.29	7.22	0.46	58.23
22/09/19	23.09	8.26	35.16	54.09	35.80	0.02	298.07	93.27	6.50	52.12
11/10/19	22.81	8.32	35.10	54.00	35.73	0.02	281.48	35.81	2.51	51.74
28/11/19	22.37	8.04	35.51	54.64	36.22	0.02	211.88	15.88	1.12	51.90

**Table 3:** Flexural strength and uniaxial compressive strength measured for mortar specimens. * Values given by the producers, n.a. = data not available.

	Flexural Strength 28 Days (MPa)	Uniaxial Compressive Strength 28 Days (MPa)	Uniaxial Compressive Strength 200 Days (MPa)
SA	2.4 ± 0.5	4.6 ± 0.9	4.8 ± 0.9
SA/Mg	2.0 ± 0.1	3.8 ± 0.1	3.9 ± 0.1
VM	3.8 ± 0.3	7.1 ± 0.8	7.5 ± 0.9
VM/Mg	3.2 ± 0.3	5.5 ± 0.2	5.8 ± 0.2
SA *	n.a.	1.88	n.a.
VM *	>1.5	>8	n.a.

**Table 4:** Results of colorimetric analysis.

	28 Days of Immersion			200 Days of Immersion						
	L	a	b	L	a	b	ΔL*	Δa*	Δb*	ΔE
SA	75.5 ± 2.2	0.4 ± 0.1	4.8 ± 0.8	76.6 ± 3.8	0.2 ± 0.1	5.0 ± 1.1	1.1	−0.2	0.3	1.1 ± 0.4
VM	80.8 ± 1.7	0.2 ± 0.1	5.1 ± 0.9	79.3 ± 2.9	0.1 ± 0.1	5.3 ± 1.5	−1.5	−0.1	0.2	1.5 ± 0.3
SA/Mg	77.7 ± 2.5	−0.1 ± 0.1	5.8 ± 0.7	78.2 ± 3.1	0.0 ± 0.1	5.3 ± 1.7	0.5	0.1	−0.5	0.7 ± 0.3
VM/Mg	76.9 ± 1.8	−0.1 ± 0.1	5.5 ± 0.6	77.1 ± 2.7	−0.1 ± 0.1	4.7 ± 1.9	0.2	0.0	−0.8	0.8 ± 0.3

**Table 5:** Results of quantitative X-ray analysis.

Sample Code	Cal	Qz	Anl	Lct	Cpx	Pl	Mca	Hl	LOAP
SA (Control test)	33.0	5.0	3.1	7.2	16.7	3.3	0.1	−	31.6
SA 3 Months	33.6	4.1	3.6	6.5	17.0	3.5	0.1	tr	31.5
SA 6 Months	30.6	4.8	3.8	7.2	17.6	4.1	0.1	1.1	30.7
SA 12 Months	33.4	5.6	4.3	7.4	18.1	6.2	0.1	tr	24.8
SA/Mg 3 Months	33.5	4.7	3.7	7.3	18.3	3.4	0.2	0.22	28.6
SA/Mg 6 Months	31.5	5.7	4.3	6.3	18.1	4.5	0.2	0.18	29.3
SA/Mg 12 Months	28.4	4.9	3.9	6.9	18.5	3.9	0.2	tr	33.3
VM (Control test)	31.5	13.0	4.4	8.4	19.4	6.7	0.1	−	16.0
VM 3 Months	31.6	10.5	4.4	7.5	19.0	5.6	0.2	0.54	21.0
VM 6 Months	31.7	5.0	4.4	7.4	18.6	4.6	0.2	−	28.2
VM/Mg 6 Months	30.2	5.2	4.0	7.3	18.0	4.9	0.1	tr	30.2

Legend: Cal, Calcite; Qz, Quartz; Pl, Plagioclase; Cpx, Clinopyroxene; Lct, leucite; Anl, Analcime; Mca, Mica; Hl, halite; Am, Amorphous phases; tr, traces; − not detected.

**Table 6:** Simultaneous Thermal Analysis TG-DTG-DSC.

Samples Code	Dehydration			Dehydration of Phyllosilicates and Decomposition of Organic Substance			Decomposition of Carbonates			Polymorphic Transformation and Sintering			R.M. (%)	Cal (%)
	40–200 °C			200–600 °C			600–850 °C			>850 °C				
	ΔW (%)	DTG (°C)	DSC (^a^) (°C)	ΔW (%)	DTG (°C)	DSC (^a,b^) (°C)	ΔW (%)	DTG (°C)	DSC (^a^) (°C)	ΔW (%)	DTG (°C)	DSC (^a,b^) (°C)		
VM (Control test)	2.36	99.4	97.3 ^a^	3.58	427.9	423.3 ^a^	11.32	780.4	772.4 ^a^	0.09	-	868 ^a^	82.65	25.70
VM (3 months)	2.25	100.1	92.2 ^a^	4.12	432.1	421.1 ^a^–567.0 ^a^	11.37	753.6	754.9 ^a^	0.94	897.2	-	81.32	25.81
VM (6 months)	3.98	98.7	98.2 ^a^	7.03	436.5–561.5	423.0 ^a^–553.1 ^a^	8.97	740.5	743.9 ^a^	1.14	926.9	-	78.88	20.36
VM/Mg (6 months)	4.66	101.5	99.6 ^a^	7.73	440.5–535.5	430.1 ^a^–536.6 ^a^	8.67	734.7	741.9 ^a^	1.28	928.8	-	77.66	19.68
SA (Control test)	2.34	112.3	107.5 ^a^	4.23	441.7	447.8 ^a^	12.94	790.7	790.7 ^a^	0	-	-	80.49	29.37
SA (3 months)	3.89	106.9	102.4 ^a^	8.08	450	413.0 ^a^	8.41	661.8–721.8	651.0 ^a^–730.0 ^a^	1.83	973.4	-	77.79	19.09
SA (6 months)	3.87	96.3	96.1 ^a^	7.68	442	444.9 ^a^	9.28	736.6	737.3 ^a^	1.8	902.6–1024.6	-	77.37	21.07
SA (12 months)	4.4	108.3	98.7 ^a^	6.79	444.8	447.9 ^a^	8.89	725.7	735.4 ^a^	1.22	910.7	-	78.70	20.18
SA/Mg (3 months)	4.18	106.7	110.4 ^a^	7.21	442.1–591.3	433.0 ^a^–543.8 ^a^	9.83	758.6	612.0 ^a^–759.8 ^a^	1.26	1057.3	-	77.52	22.31
SA/Mg (6 months)	4.51	94.4	95.3 ^a^	7.2	432.8	422.8 ^a^–547.5 ^a^	9.39	741	741.9 ^a^	1.05	913.4	-	77.85	21.32
SA/Mg (12 months)	4.88	103.1	107.9 ^a^	7.19	441.7–591.9	432.8 ^a^–526.1 ^a^–584.8 ^a^	8.25	755	754.9 ^a^	1.19	961.5	-	78.49	18.73

Legend: (^a^) = endothermic; (^b^) = esothermic; ΔW = weight loss; R.M. = residual mass; Cal = calcite; deh. = dehydroxylation; dec. = decomposition.
